# Supplementary material for: Challenges and strategies in the soluble expression of CTA1-(S14P5)4-DD and CTA1-(S21P2)4-DD fusion proteins as candidates for COVID-19 intranasal vaccines
Source: PLoS One. 2024 Dec 26;19(12):e0306153. doi: 10.1371/journal.pone.0306153 (PMC11670946; doi:10.1371/journal.pone.0306153)
Supplement: S1 Data — (DOCX) [file pone.0306153.s001.docx]

**S1 Data**

**Amino-acid sequence for CTA1-(S14P5)4-DD and CTA1-(S21P2)4-DD**

**>CTA1-(S14P5)4-DD**

MNDDKLYRADSRPPDEIKQSGGLMPRGQSEYFDRGTQMNINLYDHARGTQTGFVRHDDGYVSTSISLRSAHLVGQTILSGHSTYYIYVIATAPNMFNVNDVLGAYSPHPDEQEVSALGGIPYSQIYGWYRVHSGVLDEQLHRNRGYRDRYYSNLDIAPAADGYGLAGFPPEHRAWREEPWIHHAPPGCGNAPRSSGGGSTESNKKFLPFQQFGRDIADGGGSTESNKKFLPFQQFGRDIADGGGSTESNKKFLPFQQFGRDIADGGGSTESNKKFLPFQQFGRDIADGGGSADAQQNNFNKDQQSAFYEILNMPNLNEAQRNGFIQSLKDDPSQSTNVLGEAKKLNESQAPKADAQQNNFNKDQQSAFYEILNMPNLNEAQRNGFIQSLKDDPSQSTNVLGEAKKLNESQAPKHHHHHH

**>CTA1-(S21P2)4-DD**

MNDDKLYRADSRPPDEIKQSGGLMPRGQSEYFDRGTQMNINLYDHARGTQTGFVRHDDGYVSTSISLRSAHLVGQTILSGHSTYYIYVIATAPNMFNVNDVLGAYSPHPDEQEVSALGGIPYSQIYGWYRVHSGVLDEQLHRNRGYRDRYYSNLDIAPAADGYGLAGFPPEHRAWREEPWIHHAPPGCGNAPRSSGGGSDPSKPSKRSFIEDLLFNKVTGGGSDPSKPSKRSFIEDLLFNKVTGGGSDPSKPSKRSFIEDLLFNKVTGGGSDPSKPSKRSFIEDLLFNKVTGGGSADAQQNNFNKDQQSAFYEILNMPNLNEAQRNGFIQSLKDDPSQSTNVLGEAKKLNESQAPKADAQQNNFNKDQQSAFYEILNMPNLNEAQRNGFIQSLKDDPSQSTNVLGEAKKLNESQAPKHHHHHH

Note:

The amino acid sequences provided here are for the constructs used in the study, specifically the CTA1-(S14P5)4-DD and CTA1-(S21P2)4-DD fusion proteins. The color coding of the amino acid symbols corresponds to the 3D structures shown in Figure 1.
