# Supplementary figures and images for: Challenges and strategies in the soluble expression of CTA1-(S14P5)4-DD and CTA1-(S21P2)4-DD fusion proteins as candidates for COVID-19 intranasal vaccines
Source: PLoS One. 2024 Dec 26;19(12):e0306153. doi: 10.1371/journal.pone.0306153 (PMC11670946; doi:10.1371/journal.pone.0306153)

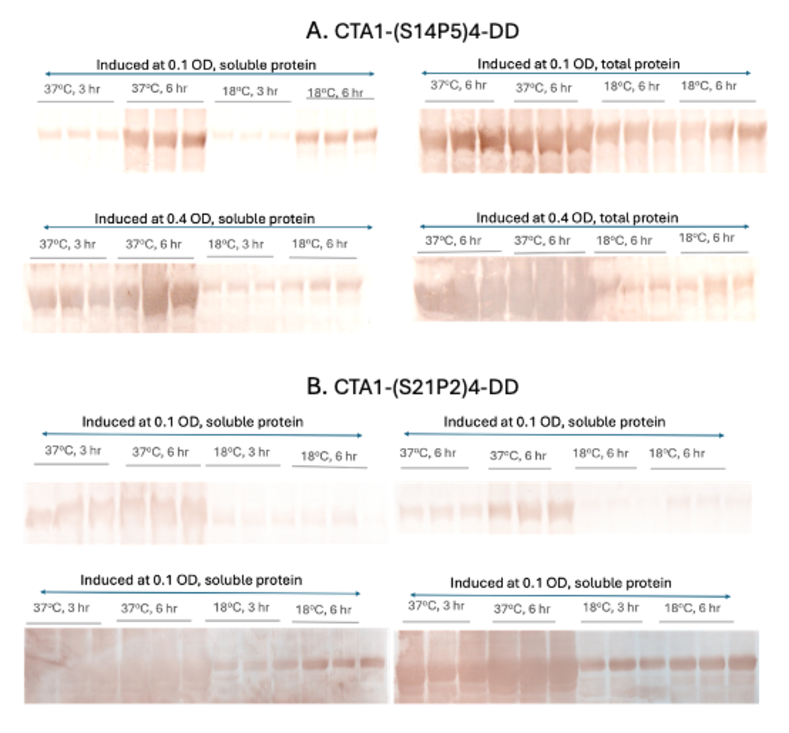

Supplement: S1 Fig — Visualization of CTA1-(S14P5)4-DD (A) and CTA1(S21P2)4-DD (B) proteins using specific antibodies in immunoblot assays. Proteins were analyzed as either soluble fractions or total proteins within bacterial cells. Cultures were grown at 37°C or 18°C, incubated for 3 or 6 hours, and induced with IPTG at early log-phase (OD600 of 0.1) or mid-log-phase (OD600 of 0.4). Note that the samples for the soluble fractions were derived from four times more bacterial cells than those for total protein. (TIF) [file pone.0306153.s002.tif]

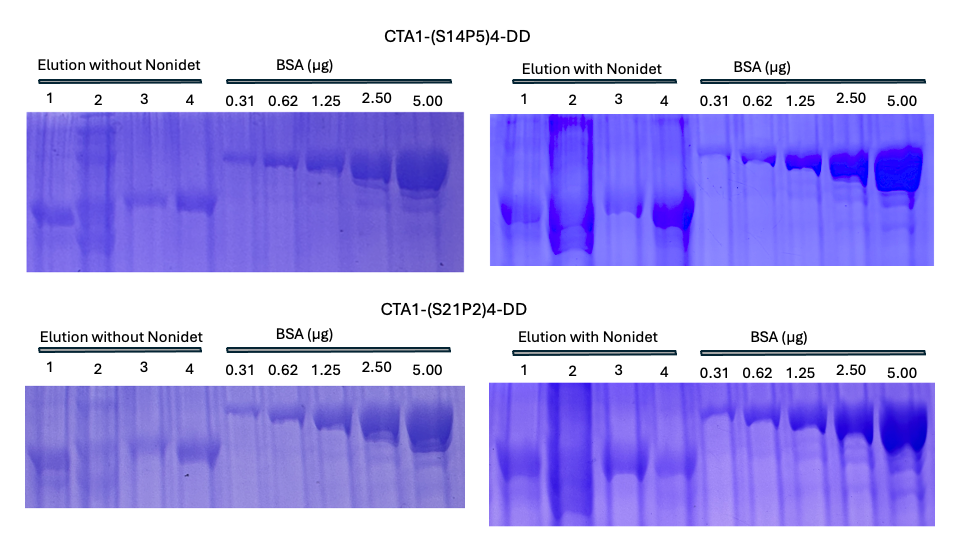

Supplement: S2 Fig — Bacterial cell lysates were prepared in PBS containing 0.1% Nonidet P40 and subjected to NiNTA column chromatography. Proteins adsorbed by the column were eluted with either 0.5 M imidazole (A, C) or 0.5 M imidazole containing 0.1% Nonidet P40. Lane 1: Proteins from 50 μL culture after induction. Lane 2: Proteins from the supernatant of cell lysate from 125 μL culture. Lane 3: Eluate from the NiNTA column derived from 500 μL culture. Lane 4: Dialyzed and concentrated eluate from 2 mL culture. (TIF) [file pone.0306153.s003.tif]
