# Supplementary material for: Challenges and strategies in the soluble expression of CTA1-(S14P5)4-DD and CTA1-(S21P2)4-DD fusion proteins as candidates for COVID-19 intranasal vaccines
Source: PLoS One. 2024 Dec 26;19(12):e0306153. doi: 10.1371/journal.pone.0306153 (PMC11670946; doi:10.1371/journal.pone.0306153)
Supplement: S1 Table — (DOCX) [file pone.0306153.s004.docx]

S1 Table. Percentage of soluble protein recombinants at different incubation temperatures, durations of incubation, and growth stages of IPTG induction.

| Protein Recombinants | Incubation Temperature | OD_600_ induction and duration of incubation | | | |
| --- | --- | --- | --- | --- | --- |
|  |  | 0.1 OD_600_ | | 0.4 OD_600_ | |
|  |  | 3 hr | 6 hr | 3 hr | 6 hr |
| CTA1-(S14P5)4-DD | 18^o^C | 2 ± 0 % | 16 ± 3 % | 27 ± 7 % | 23 ± 3 % |
|  | 37^o^C | 2 ± 1 % | 21 ± 2 % | 25 ± 3 % | 52 ± 9 % |
| CTA1-(S21P2)4-DD | 18^o^C | 3 ± 0 % | 6 ± 1 % | 2 ± 1 % | 4 ± 1 % |
|  | 37^o^C | 16 ± 2 % | 46 ± 5% | 5 ± 1 % | 27 ± 2 % |

**Note:** The percentage of soluble proteins was significantly higher (p<0.01) at an incubation temperature of 37°C and a duration of incubation of 6 hours for both proteins, and IPTG induction at 0.4 OD_600_ for CTA1-(S21P2)4-DD.
