# Supplementary material for: Challenges and strategies in the soluble expression of CTA1-(S14P5)4-DD and CTA1-(S21P2)4-DD fusion proteins as candidates for COVID-19 intranasal vaccines
Source: PLoS One. 2024 Dec 26;19(12):e0306153. doi: 10.1371/journal.pone.0306153 (PMC11670946; doi:10.1371/journal.pone.0306153)
Supplement: S2 Table — (DOCX) [file pone.0306153.s005.docx]

S2 Table. Production of soluble CTA1-(S14P5)4-DD, as indicated by different cultivation temperatures, incubation times, and growth stages of induction

| **Cultivation Temperature** | **Incubation Time** | **OD_600_  Induction** | |
| --- | --- | --- | --- |
|  |  | **0.1** | **0.4** |
| 37^o^C | 3 hr | 676* | 4246 |
|  |  | 704 | 3914 |
|  |  | 702 | 2666 |
|  | 6 hr | 4748 | 4310 |
|  |  | 4651 | 7078 |
|  |  | 5462 | 5694 |
| 18^o^C | 3 hr | 254 | 593 |
|  |  | 280 | 948 |
|  |  | 355 | 728 |
|  | 6 hr | 2275 | 1160 |
|  |  | 2291 | 1432 |
|  |  | 2213 | 1415 |

Note:

(*) Peak area or density of band identified by specific antibody in immunoblot assay (S1 Fig)

Wilcoxon's significant difference test:

18°C<37°C: *p<*0.05

3 hr < 6 hr: *p<*0.05,

0.1 OD_600_ > 0.4 OD_600_ *p*>0.05
