# Supplementary material for: Challenges and strategies in the soluble expression of CTA1-(S14P5)4-DD and CTA1-(S21P2)4-DD fusion proteins as candidates for COVID-19 intranasal vaccines
Source: PLoS One. 2024 Dec 26;19(12):e0306153. doi: 10.1371/journal.pone.0306153 (PMC11670946; doi:10.1371/journal.pone.0306153)
Supplement: S3 Table — (DOCX) [file pone.0306153.s006.docx]

S3 Table. Production of Soluble CTA1-(S21P2)4-DD, as indicated by at Different Cultivation Temperatures, Incubation Times, and Growth Stages of Induction

| **Cultivation Temperature** | **Incubation Time** | **OD_600_  Induction** | |
| --- | --- | --- | --- |
|  |  | **0.1** | **0.4** |
| 37^o^C | 3 hr | 3876* | 3889 |
|  |  | 5586 | 2774 |
|  |  | 5844 | 2222 |
|  | 6 hr | 7572 | 6659 |
|  |  | 8098 | 7265 |
|  |  | 9218 | 8440 |
| 18^o^C | 3 hr | 1925 | 415 |
|  |  | 1753 | 480 |
|  |  | 1547 | 429 |
|  | 6 hr | 1824 | 1819 |
|  |  | 2788 | 1319 |
|  |  | 566 | 1112 |

Note:

(*) Density of band identified by specific antibody in immunoblot assay (S1 Fig)

Wilcoxon's significant difference test:

18°C <37°C: *p*<0.05;

3 hr < 6 hr: *p<*0.05,

0.1 OD_600_ > 0.4 OD_600_ *p*<0.05
