# Supplementary material for: Challenges and strategies in the soluble expression of CTA1-(S14P5)4-DD and CTA1-(S21P2)4-DD fusion proteins as candidates for COVID-19 intranasal vaccines
Source: PLoS One. 2024 Dec 26;19(12):e0306153. doi: 10.1371/journal.pone.0306153 (PMC11670946; doi:10.1371/journal.pone.0306153)
Supplement: Attachment — Submitted filename: Response to Reviewers.docx [file pone.0306153.s007.docx]

**Dear Dr. Haitham Mohamed Amer,**

We sincerely appreciate the opportunity to revise and resubmit our manuscript titled "Optimization of Soluble Expression of CTA1-(S14P5)4-DD and CTA1-(S21P2)4-DD Fusion Proteins as Candidates for COVID-19 Intranasal Vaccines" (PONE-D-24-23892). We are grateful for the constructive feedback provided by the reviewers and the academic editor. We have carefully considered all comments and have made the necessary revisions to address each point raised.

In response to the editor's specific requests:

1. **Manuscript Style Compliance:** We have ensured that the manuscript meets PLOS ONE's style requirements, including file naming conventions. The revisions have been made in accordance with the PLOS ONE formatting guidelines.
2. **Role of Funders:** We have clearly stated in the manuscript that "The funders had no role in study design, data collection and analysis, decision to publish, or preparation of the manuscript." This statement has been included in the revised manuscript.
3. **Figure Improvements:** We have addressed the issue regarding the clarity of our figure images by using the web-based imaging review tool, PACE. The revised figures have been uploaded, and we kindly ask the editor to review them to ensure they now meet the required standards. If further improvements are necessary, we are happy to make additional adjustments.
4. **Reviewer Comments:** We have carefully addressed each of the reviewers' comments, making significant revisions to the manuscript where necessary. Our detailed responses to each comment are attached to this letter.
5. **Submission of Revised Manuscript:** We have uploaded the revised manuscript with track changes, an unmarked version of the manuscript, and our detailed response to the reviewers as separate files as requested.

We hope that the revisions and the additional information provided satisfactorily address the concerns raised. We believe that these improvements enhance the clarity and scientific rigor of our work.

Thank you for considering our revised manuscript for publication. We look forward to your feedback.

**Sincerely,**

Simson Tarigan
sitariganta@gmail.com
National Research and Innovation Agency of Indonesia

**Response to Reviewers**

**Reviewer 1**

**ABSTRACT AND INTRODUCTION**

1. The introduction effectively establishes the rationale for developing intranasal vaccines against COVID-19. It provides a good overview of the advantages of intranasal vaccination and the potential of CTA1-DD fusion proteins as mucosal adjuvants. The authors successfully contextualize their work within the broader field of vaccine development. However, **the transition to the specific aims of this study could be more clearly articulated.**

**Our response:** Thank you for your insightful feedback. We appreciate your positive comments regarding the rationale and contextualization provided in the introduction. In response to your suggestion, we have made edits to the abstract and the introduction to better articulate the specific aims of our study.

While we have endeavored to address this comment as thoroughly as possible, we acknowledge that the transition to the specific aims of the study may still not fully meet your expectations. We have emphasized the construction and characterization of the fusion proteins CTA1-(S14P5)4-DD and CTA1-(S21P2)4-DD, focusing on their solubility and functionality, as well as potential challenges in the expression and purification processes.

**MATERIALS AND METHODS**

2.1 The exact sequences of the constructs are not provided, which is essential for reproducibility

**Our response:** We appreciate the reviewer’s comment on the importance of providing the exact sequences for reproducibility. To address this concern, we have included the exact sequences of the constructs used in our study as supplementary data (S1 Data). Additionally, we have clarified this in the manuscript with the following statement: “The CTA1-(S14P5)4-DD and CTA1-(S21P2)4-DD constructs were generated by fusing the catalytic subunit of cholera toxin (CTA1) at the N-terminal end of the tandem repeat SARS-CoV-2 epitopes S14P5 or S21P2 and the two-domain D of the staphylococcal protein A at the C-terminal end (Fig 1A, S1 Data).”

2.2 The specific *E. coli* strain used for expression is not mentioned

**Response:** We appreciate reviewer’s attention to detail. The *E. coli* strain we used was BL21(DE3) (Thermo Fisher Scientific). We have clarified this in the manuscript. The updated text in the Materials and Methods section now reads: “The plasmids were transformed into a competent **BL21(DE3) strain of *E. coli* (Thermo Fisher Scientific)**.”

2.3 The protein quantification method using densitometry needs more detailed description.

**Our response:** We appreciate the reviewer's insightful comment regarding the protein quantification method using densitometry. We have clarified this methodology by adding the following detailed description to the Materials and Methods section:

"Protein concentrations were assessed by densitometry of protein bands on SDS-PAGE gels or immunoblots using ImageJ, a public domain program from the National Institutes of Health (NIH, USA). The images of the SDS-PAGE and immunoblots were imported into ImageJ and converted to 32-bit format. Background subtraction was performed to enhance accuracy. A rectangle was drawn around the protein bands, and a plot profile of each band was generated. A line was then drawn to connect the base of each protein peak, and the area under the curves was measured. Special precautions were taken to ensure the accurate delineation of the peak bases, thus providing true peak areas. These peak areas correspond to the grayscale intensity of the bands, which directly correlates with the amount of protein present. The absolute concentrations of proteins were determined using a standard curve constructed with BSA (Bovine Serum Albumin) at concentrations of 0, 0.31, 0.62, 1.25, 2.5, and 5.0 µg/mL, which were loaded on the same gel."

2.4. Statistical analysis methods are mentioned but not fully explained.

**Our response:** We appreciate the reviewer's insightful comment and have expanded the description of the statistical analysis methods in the Materials and Methods section to provide more detail. The updated section now reads:

**Statistical Analysis**

Descriptive statistics were used to summarize the protein expression levels or concentration data. Wilcoxon rank-sum test was employed to compare protein concentrations between different experimental conditions. This non-parametric test is suitable for small sample sizes or data that does not assume a normal distribution. A *p*-value less than 0.05 was considered statistically significant, indicating a significant difference between protein concentrations under different conditions. All statistical analyses were carried out using Jamovi, an open-source statistical software (<https://www.jamovi.org>).

**RESULT**

3.1 More quantitative data presentation, particularly in comparing soluble protein yields under different conditions.

**Our response:** Thank you for your valuable feedback. We appreciate your suggestion for more quantitative data presentation. In response, we have made changes to the manuscript to include more detailed quantitative data in comparing soluble protein yields under different conditions. Below is the updated content in the manuscript:

**Effect of growth stage (OD_600_) at induction, temperature and duration of incubation**

Contrary to the in silico prediction, both CTA1-(S14P5)4-DD and CTA1-(S21P2)4-DD were poorly soluble when overexpressed in E. coli. The soluble fractions constituted only a negligible portion of the total proteins, and exact quantification was often unreliable due to smearing observed in many protein bands (S1 Fig, S1 Table). Lowering the culture temperature from 37°C to 18°C did not increase protein solubility (p>0.05). Quantitative data showed that lowering the culture temperature from 37°C to 18°C decreased the soluble protein yield for CTA1-(S14P5)4-DD from 10% to 5% and for CTA1-(S21P2)4-DD from 12% to 6%. Prolonging the incubation time from 3 to 6 hours increased the soluble protein yield significantly, with CTA1-(S14P5)4-DD rising from 8% to 15% and CTA1-(S21P2)4-DD from 9% to 18%. Additionally, induction at an early log phase (OD_600_ of 0.1) resulted in higher production of soluble CTA1-(S21P2)4-DD (17%) compared to an OD_600_ of 0.4 (12%). However, a significant increase by induction at the early log phase was not observed in the production of soluble CTA1-(S14P5)4-DD (p>0.05) (S2 and S3 Tables).

**Effect of detergent in the extraction and purification buffer**

Protein purification using the native buffer (0.5 M NaCl, 0.1 M sodium phosphate, pH 9) with the NiNTA purification system yielded low amounts of CTA1-(S14P5)4-DD and even lower amounts of CTA1-(S21P2)4-DD. Adding detergents to the native buffer significantly increased the yields. For CTA1-(S14P5)4-DD, Nonidet P40 at 0.01% increased yields by 5 times, at 0.05% and 0.1% by 7 times over the native buffer. Triton X-100 at 0.01%, 0.05%, and 0.1% increased yields by 5, 6, and 7 times, respectively. Tween 20 was less effective, with 0.01% yielding a 2 times increase, and both 0.05% and 0.1% yielding a 5 times increase over the native buffer.

The increase in soluble yields was more profound with CTA1-(S21P2)4-DD. Nonidet P40 at 0.01% increased yields by 7 times, at 0.05% by 13 times, and at 0.1% by 16 times over the native buffer. Triton X-100 at 0.01%, 0.05%, and 0.1% increased yields by 8, 11, and 8 times, respectively. Tween 20 at 0.01% yielded a 2-fold increase, and at both 0.05% and 0.1% yielded an 11-fold increase over the native buffer (Fig 3).

3.2 Clearer explanation of the discrepancies between small-scale and large-scale purification results

**Our response:** Thank you for your valuable feedback. To briefly explanation the discrepancies between small-scale and large-scale purification results, we have added the following explanation to the Results section:

" The discrepancy in yields between the small and larger volumes can be attributed to several factors. At small scales, higher mixing efficiency during bacterial cultivation, more efficient cell sonication, and much shorter purification times were achieved compared to larger scales. In contrast, larger-scale processes face challenges in maintaining consistent mixing, efficient sonication, and timely purification, leading to variations in protein yield and purity. Further optimization of these parameters is required to improve the efficiency and scalability of large-scale purifications."

3.3 More consistent application of statistical analyses to support the conclusions drawn. The figures are generally informative, but some, particularly those showing gel images, could be improved in terms of clarity and labeling.

**Our response:** Thank you for your valuable feedback. We acknowledge that statistical analysis was not applied uniformly across all experiments. Specifically, we used the Wilcoxon rank-sum test in the "Effect of growth stage (OD_600_) at induction, temperature, and duration of incubation" experiments. However, in the "Effect of detergent in the extraction and purification buffer" experiment, statistical analysis was not carried out due to the lack of replication of samples. Despite this, the profound increase in the yields of soluble proteins by the addition of detergents indicates their significance.

To address your concern, we have added the following statement at the end of the relevant section: “Statistical analysis was not carried out because no replication of the samples; nevertheless, the profound increase in the yields of soluble proteins by the addition of the detergents should indicate their significance.”

**DISCUSSION**

4.1 More critical analysis of why the *in silico* predictions of solubility were inaccurate.

**Our response:** Thank you for your comment. We appreciate the need for a more critical analysis of the discrepancies between in silico predictions and actual solubility results. We have expanded the paragraph to address this issue more comprehensively:

The solubility of CTA1-(S14P5)4-DD and CTA1-(S21P2)4-DD on overexpression was predicted by two computational tools: (1) SoluPro, which predicts solubility based on amino acid composition, dipeptide composition, and physicochemical properties, employing a machine learning approach, and (2) NetSolP, which predicts solubility based on amino acid composition, secondary structure, and solvent accessibility employing a neural network-based approach. These tools have undergone significant improvements, achieving accuracies of 74% for SOLUPro and 70% for NetSolP, surpassing many previous methods [29, 30]. However, despite these advancements, there remains a risk of misprediction, particularly for engineered proteins like CTA1-(S14P5)4-DD and CTA1-(S21P2)4-DD, as these computational tools may not have been trained on similar proteins. Furthermore, in silico tools often do not account for complex cellular environments, such as expression levels, folding mechanisms, chaperone interactions, and post-translational modifications, which can significantly influence solubility [31; 32]. The propensity for aggregation, which is a critical factor in protein solubility, is also challenging to predict accurately [33; 34]. To improve solubility predictions, future computational tools should incorporate more diverse training datasets and consider the cellular context and experimental conditions.

4.2 "Deeper exploration of alternative strategies to improve solubility and yield."

**Our response:** Thank you for your valuable feedback. We recognize the importance of exploring alternative strategies to enhance the solubility and yield of recombinant proteins. In response, we have expanded the discussion by adding the following:

“There are several alternative strategies that can be exploited to improve the solubility and yield of CTA1-(S14P5)4-DD and CTA1-(S21P2)4-DD fusion proteins. Practical approaches include incorporating chemical additives like arginine, glycerol, betaine, and trehalose in the growth medium or purification buffers, which can stabilize proteins and inhibit aggregation [44]. Additionally, co-expressing molecular chaperones such as GroEL/GroES, DnaK/DnaJ/GrpE, or trigger factor can assist in proper protein folding and prevent aggregation [45]”.

4.3 Clearer articulation of the next steps needed to advance these candidates toward vaccine development.

**Our response:** Thank you for your valuable suggestion. In response, we have expanded the discussion by adding the following sentences at the end of the discussion:

"To advance these candidates toward vaccine development, several critical steps are necessary. First, in vivo immunogenicity and efficacy studies need to be conducted in appropriate animal models. Second, optimizing the expression and purification protocols to increase yield and solubility will be essential. Finally, detailed safety and toxicity assessments are required to ensure the candidates are safe for use in humans."

4.4 Discussion of the potential immunogenicity and protective efficacy of these constructs, even if not experimentally addressed in this study

**Our response:** Thank you for your valuable comment. We acknowledge the reviewer's comment regarding the discussion of the potential immunogenicity and protective efficacy of the constructs. We have indeed addressed this in the first paragraph of the Discussion section. In this study, we constructed two fusion proteins, CTA1-(S14P5)4-DD and CTA1-(S21P2)4-DD, as candidates for intranasal vaccines against SARS CoV-2. Each protein contains a linear epitope S14P5 or S21P2 of the SARS CoV-2 spike protein, which has been shown to be efficacious in inducing neutralizing antibodies. The linear epitopes were made in the form of four tandem repeats. Our previous study confirmed that the S14P5 and S21P2 in this form enhanced the immunogenicity of the epitopes, and the induced antibodies effectively recognized the SARS-CoV-2 virus. To further enhance the immune responses and optimize them as intranasal vaccines, each of the tandem repeat epitopes was conjugated to the catalytic subunit of cholera toxin (CTA1) and the domain D of staphylococcal protein A in dimer form (DD). The choice of domain D was beneficial due to its ability to bind not only IgG but also IgA and IgM.

**Response to Reviewers**

**Reviewer # 2**

1. First, the title is not very precise, as in fact no optimization was really achieved.

**Our response:** Thank you for your insightful feedback regarding the title of our manuscript. We agree that the title should accurately reflect the content and findings of our study. While our work identified potential strategies and challenges in the soluble expression of the fusion proteins, full optimization was not achieved. Therefore, we propose the following revised title: **"Challenges and strategies in the soluble expression of CTA1-(S14P5)4-DD and CTA1-(S21P2)4-DD fusion proteins as candidates for COVID-19 intranasal vaccines"**

2. Although the abstract and title bring the conceptually correct term “fusion protein”, on pages 3 (L. 89-99) and 4 (L. 119) the authors use conjugation or conjugating. Normally, in the vaccine field, conjugation refers to a chemical reaction to bind two molecules and is largely applied to conjugate vaccines, in which polysaccharides are chemically bound to a carrier protein. Therefore, the terms conjugation or conjugating should be replaced by fusion or fusing (also fused) to avoid misunderstandings. Also, it is misleading to call a 150 mL culture (information found only in Fig S2 caption) a scale-up experiments, since the term “scale-up” brings at least the idea of bioreactor cultivation.

**Our response:** Thank you for your valuable comments. We agree with your suggestions and have made the necessary changes to the manuscript. The terms "conjugation" and "conjugating" have been replaced with "fusion" and "fusing" to accurately reflect the nature of the fusion proteins. Additionally, the reference to "scale-up experiments" has been clarified to "larger volume experiments" to avoid any misconceptions, specifying that the experiments involved 150 mL cultures, which is not indicative of bioreactor-scale cultivation. These adjustments should enhance the clarity and accuracy of our manuscript.

3. The authors affirm that “the vaccine candidates are expected be thermostable because they are based on linear epitopes” (p. 4, L. 108-109). This statement related two independent facts without scientific basis, there is no reason for linear epitopes be thermostable or conformational epitopes be labile. The stability of a protein is determined by a series of physical-chemical characteristics and presence of protease cleavage sites rather than if they are composed of linear epitopes.

**Our response:** We agree with the reviewer that the stability of a protein is determined by a series of physicochemical characteristics and the presence of protease cleavage sites. However, our expectation for the thermostability of vaccines based on linear epitopes is due to the fact that linear epitopes are composed of continuous amino acid sequences, making them less sensitive to conformational changes induced by temperature fluctuations. Unlike conformational epitopes, which consist of discontinuous segments reliant on specific spatial arrangements, are more susceptible to structural changes caused by temperature variations, potentially affecting their recognition by antibodies. Therefore, linear epitopes are generally considered more thermostable compared to conformational epitopes

4. The authors claim that the approach of this study could be readily applied to other infectious disease (p. 4, L. 114- 115), using linear epitopes that induce neutralizing antibodies (p. 15, L. 437-445). While it might be true that one can use bioinformatics to design similar fusion molecules simply changing the epitopes, as each novel protein will present distinct physical-chemical characteristics that will influence the production and purification processes, all new constructs will need a completely new process development to really generate a vaccine candidate. Even from the immunological point of view, it is hard to say that all new fusion proteins will induce neutralizing antibodies when the epitopes were presented in the proposed CTA1-epitope-DD format. This manuscript limits to show the attempts for production and purification of two fusion proteins. Although recognized by antibodies in immunoblot assays, there is no guarantee that they will induce neutralizing antibodies.

**Our response:** We appreciate the reviewer's insights and understand the concerns regarding the application of our approach to other infectious diseases. While it is true that each novel fusion protein will present distinct physical-chemical characteristics that influence production and purification processes, we have based our expectations on established principles and previous findings.

Firstly, as mentioned earlier, linear epitopes are composed of continuous amino acid sequences, making them less sensitive to conformational changes. This intrinsic stability supports our expectation that linear epitopes will remain stable when fused with stable proteins like CTA1 and DD. The stability of the CTA1 and DD components has been well-documented, further supporting this assumption.

Secondly, if a linear epitope has been shown to elicit neutralizing antibodies, it is reasonable to expect that this capability will be preserved when the epitope is fused with CTA1-DD. The role of CTA1 as a mucosal adjuvant and DD in targeting immune cells should enhance, not hinder, the immunogenicity of the linear epitopes.

Lastly, while each fusion protein will require specific optimization for production and purification, the foundational concept remains applicable. The use of computational tools to design and predict epitopes and the general strategies for expression and purification can be adapted and refined for different targets.

5. It is difficult to follow the authors analysis of Fig. 1 related to the similarities between cryo-electron microscopy and predicted structures of the epitopes. Maybe a superimposed image showing both structures would be better to support the conclusions.

**Our response:** We appreciate the reviewer's suggestion to improve the clarity of Figure 1. While we understand the potential value of a superimposed image, we believe that a detailed explanation in the figure legend can sufficiently convey the similarities between the cryo-electron microscopy (cryo-EM) structures and the predicted structures by AlphaFold2. We have revised the figure legend to provide a clearer description of the comparison.

**Revised Figure Legend:**

**Fig 1. Structural comparison of predicted and cryo-electron microscopy (cryo-EM) structures of the fusion proteins and SARS-CoV-2 spike protein epitopes.**

(A) **Schematic representation of the fusion proteins CTA1-(S14P5)4-DD and CTA1-(S21P2)4-DD, illustrating the arrangement of the CTA1, tandem repeat epitopes, and DD domains.**

(B) **Predicted tertiary structure of CTA1-(S14P5)4-DD by AlphaFold2, visualized using ChimeraX. CTA1 is colored in blue, (S14P5)4 in red, and DD in violet.**

(C) **Cryo-EM structure of the SARS-CoV-2 spike protein (PDB # 6VXX), highlighting the secondary structure of the linear epitope S14P5 in red.**

(D) **Predicted tertiary structure of CTA1-(S21P2)4-DD by AlphaFold2, visualized using ChimeraX. CTA1 is colored in blue, (S21P2)4 in red, and DD in violet.**

(E) **Cryo-EM structure of the SARS-CoV-2 spike protein (PDB # 6VXX), highlighting the secondary structure of the linear epitope S21P2 in red.**

**Explanation:** **The predicted structures (B and D) were generated using AlphaFold2 and depict the spatial arrangement of the fusion proteins' components. The cryo-EM structures (C and E) show the actual configurations of the S14P5 and S21P2 epitopes within the SARS-CoV-2 spike protein. The consistent coloring (red) of the epitopes in both predicted and cryo-EM structures emphasizes their structural preservation, supporting the hypothesis that the epitopes maintain their conformation when fused within the larger constructs.**

6. Some information is missing in the Material & Methods section and additional details

should be provided. Below, a list of examples:
1. Which is the culture medium used for protein production?
2. One can suppose that both recombinant proteins were obtained with His-tag because the results showing elution fractions from NiNTA column. However, no indication about His-tag location (N- or C-terminal) was given, which should be included in Material & Methods section and in Fig. 1A.

3. Details about the metal affinity chromatography should be included: which column was used (volume, supplier, etc.) and the conditions for equilibrium and elution (buffers, gradient, flow rate, etc.), as well as about the subsequent operations that were mentioned only in the Results section, i.e., elution, dialysis, and concentration in buffers without detergent.

4. Are sonication conditions at “large” scale the same as those described in L. 159-160?
5. For densitometric analysis, how total protein was measured? Was protein concentration taken into consideration for estimation of the percentage of soluble protein? A BSA standard curve was done in each SDS-PAGE? More details on how this percentage was calculated should be given.

**6.1** Which is the culture medium used for protein production?

**Our response:** We appreciate the reviewer's careful consideration of the Materials & Methods section. The culture medium used for protein production was indeed specified as Luria-Bertani (LB) broth supplemented with 30 µg/mL kanamycin. This information has been added to the "Expression of CTA1-(S14P5)4-DD and CTA1-(S21P2)4-DD" section of the manuscript.

**6.2** One can suppose that both recombinant proteins were obtained with His-tag because the results showing elution fractions from NiNTA column. However, no indication about His-tag location (N- or C-terminal) was given, which should be included in Material & Methods section and in Fig. 1A

Thank you for your insightful comment. We apologize for the oversight regarding the His-tag information. Indeed, the use of the pET30a(+) expression plasmid implies that a His-tag is added to the C-terminal of the recombinant proteins. We have updated the Materials & Methods section to specify this clearly, adding a note after 'pET30a(+) expression plasmids' to indicate the presence and location of the His-tag

Synthetic genes for CTA1-(S14P5)4-DD and CTA1-(S21P2)4-DD were generated by reverse translation of the amino acid sequences and codon optimization for prokaryotic expression. The synthetic genes were inserted into the pET30a(+) expression plasmids**, which added a His-tag to the C-terminal end of the recombinant proteins** (Genscript Inc. USA).

6.3 Details about the metal affinity chromatography should be included: which column was used (volume, supplier, etc.) and the conditions for equilibrium and elution (buffers, gradient, flow rate, etc.), as well as about the subsequent operations that were mentioned only in the Results section, i.e., elution, dialysis, and concentration in buffers without detergent.

**Our response:** Thank you for your detailed feedback regarding the metal affinity chromatography. We apologize for the oversight and appreciate the opportunity to provide additional details.

We have included the specific details about the metal affinity chromatography used in our experiments: "For experiments involving larger culture volumes, cell pellets from 150 mL cultures were resuspended in three separate 8 mL portions of native buffer, with and without the addition of 0.1% Nonidet P-40. The cell suspension was sonicated for five intervals of 30 seconds each, followed by centrifugation at 6,000 x g for 15 minutes, with the supernatant carefully collected. For small-scale purification, we used Mini-Bio Spin™ chromatography columns (Bio-Rad) with 100 µL resin and a 0.5 mL sample volume. For larger-scale purification, we employed Econo-Pac® chromatography columns (Bio-Rad) with 1 mL resin and a 24 mL sample volume. Purification was conducted according to the vendor's protocol with some modifications. The binding and washing buffer consisted of 0.5 M NaCl, 0.1 M sodium phosphate, and 30 mM imidazole at pH 9 (native buffer). The elution buffer contained 0.5 M imidazole in the native buffer. In some experiments, detergents such as Nonidet P40, Triton X-100, or Tween-20 were added to the buffers at concentrations of 0.01%, 0.05%, and 0.1%. Dialysis was carried out using 10 kDa-MWCO dialysis tubing (Sigma Aldrich) against PBS, and concentration was performed using 10 kDa-MWCO centrifugal filter units (Ultracel®-10 K, Amicon)."

6.4. Are sonication conditions at “large” scale the same as those described in L. 159-160?

**Our response:** We have clarified the sonication conditions for larger-scale experiments: "The sonication conditions for larger-scale experiments were adjusted to accommodate the increased volume. Cell pellets from 50 mL cultures were suspended in 8 mL native buffer, with and without the addition of 0.1% Nonidet P-40. The cell suspension was sonicated for five intervals of 30 seconds each, followed by centrifugation at 6,000 x g for 15 minutes to collect the supernatant."

6. 5 For densitometric analysis, how total protein was measured? Was protein concentration taken into consideration for estimation of the percentage of soluble protein? A BSA standard curve was done in each SDS-PAGE? More details on how this percentage was calculated should be given.

**Our response:** Thank you for your detailed feedback. We have revised the Materials and Methods section to provide additional clarity and address the points raised.

**Analysis of Total and Soluble Fusion Proteins**

For the analysis of total protein, 0.5 mL of bacterial culture was centrifuged at 10,000 x g for 5 minutes to pellet the cells. The resulting pellet was solubilized in 50 µL of SDS-PAGE sample buffer, and 10 µL of this suspension was loaded into a well of a 15-well polyacrylamide gel of the Mini-PROTEAN II electrophoresis apparatus (Bio-Rad). The percentage of polyacrylamide was 10% for the separating gel and 4% for the stacking gel.

To analyze soluble protein, 1 mL of bacterial culture was centrifuged at 10,000 x g for 5 minutes to pellet the cells. The pellet was resuspended in 0.5 mL of PBS containing 10 mM EDTA and sonicated for three intervals of 30 seconds each. Following sonication, the sample was centrifuged again at 10,000 x g for 5 minutes, and the supernatant was carefully collected. The proteins in the supernatant were precipitated with trichloroacetic acid according to the standard protocol [23]. The precipitated proteins were then solubilized in 50 µL of SDS-PAGE sample buffer, and 10 µL of this solution was loaded into an SDS-PAGE gel as previously described.

For the immunoblot assay, proteins from the polyacrylamide gel were transferred onto a nitrocellulose membrane, and CTA1-(S14P5)4-DD and CTA1-(S21P2)4-DD were identified respectively with rabbit-anti-(S14P5)4 and rabbit-anti-(S21P2)4 antibodies produced in our previous studies [17]. Protein concentrations were assessed by densitometry of protein bands on SDS-PAGE gel or immunoblot, using ImageJ open-source software (NIH, USA).

Protein concentrations were assessed by densitometry of protein bands on SDS-PAGE gel or immunoblot using ImageJ (NIH), a public domain program from the National Institutes of Health (USA). The images of SDS-PAGE and immunoblot were imported into ImageJ and converted to 32-bit format. After subtracting the background, a rectangle was placed around the protein bands, and a plot profile of each protein band was generated. A line was drawn to connect the base of each protein peak, and the area under the curves was obtained. Special precautions were taken to correctly connect the base of the peaks to obtain the true peak area. The peak area represents the gray intensity of the band and correlates with the amount of protein in the band. The absolute concentration of proteins was calculated from a standard curve constructed using BSA at 0, 0.31, 0.62, 1.25, 2.5, and 5.0 µg/mL, which was loaded on the same gel.

7. The effect of detergents on solubilization was empirically tested and the statement that “optimal detergent concentration appears to be around 0.1%” is very imprecise. In addition, it is in contradiction with the following sentence of the text that says: “increasing the concentration from 0.05% to 0.1% did not result in significant additional increases in soluble protein amounts”. How can 0.1% be the optimal concentration if the increasing from 0.05% to 0.1% did not change the results? Finally, it is not clear why 0.1% Nonidet-P40 was chosen as optimal condition for extraction, as the results with this detergent and Triton X-100 seemed to be similar in terms of amount of soluble protein, and which are the volumes treated in the test of different detergents (L. 255-265) and in the scaled-up process to a larger culture volume (L. 272).

**Our response:** Thank you for your insightful comments regarding the detergent concentrations and their effects on protein solubilization. We acknowledge the need for clarity and precision in presenting these results. The statement that "the optimal detergent concentration appears to be around 0.1%" was intended to reflect the highest yield obtained, but we recognize that this was imprecise and contradictory given the data presented.

We have revised the manuscript to state: "Further yield improvements were observed with higher concentrations of detergents, including Tween 20 at 0.05% and 0.1%. However, increasing the concentration from 0.05% to 0.1% did not result in significant additional increases in soluble protein amounts. Therefore, both 0.05% and 0.1% concentrations were considered effective, with no marked difference in the yield of soluble proteins between these concentrations."

Regarding the volumes treated, the small-scale tests involved 1.5 mL samples, while the larger-scale tests used 150 mL samples. This information has now been included in the revised Materials and Methods section to provide greater clarity.

8. The manuscript discusses the functionality in several passages of the text, but this work did not show any direct results that could confirm the two fusion proteins have CTA1 or DD functions preserved, or they induce neutralizing epitopes. It would be interesting to discuss also why assays to show function were not performed and what is still needed to achieve such results.

**Our response:** Thank you for your valuable comment. We agree that direct experimental confirmation of the functionality and immunogenicity of the fusion proteins is crucial. However, our current study focused primarily on the construction and initial characterization of the CTA1-(S14P5)4-DD and CTA1-(S21P2)4-DD constructs. The primary aim was to demonstrate the feasibility of expressing and purifying these constructs in E. coli, which is a critical first step.

Future work will focus on direct experimental validation of the functionality and immunogenicity of these constructs. Specifically, we plan to carry out immunization experiments in animals to determine the immunogenicity of the fusion proteins. These studies will assess whether the constructs can elicit a robust immune response, including the production of neutralizing antibodies against SARS-CoV-2.

9. **Minors**

- p. 4, L. 123, “residu132” should be “residue 132”:

**Our response:** Thank you for pointing out this error. We have corrected "residu132" to "residue 132" in the manuscript.

- L. 130-133, include DeepSoluE software that was employed for solubility prediction according to Table 1.

**Our response:** Thank you for pointing out this error. We have include the DeepSoluE software in the Materials and methods section.

- L. 141, I could not find any plasmid pET38 in any of the plasmid suppliers. The reference 17 mentioned pET30a+. There is also a pET28a+ available for cloning at GenScript website. Please, verify the correct plasmid used or describe it better in case it is a modification of some commercial vector.

**Our response:** Thank you for pointing out this error. The right plasmid is pET30a+. We have corrected

- L. 246, write E. coli in italics, please.

**Our response:** Thank you for pointing out this error. We have written *E. coli* in italics

- L. 287, which are the sonication conditions for the “large” volume experiments?
  **Our response:** Thank you for your insightful comment. The sonication conditions for the “large” volume experiments has been clarified in the Materials and Methods section. “sonicated for five intervals of 30 seconds each, followed by centrifugation at 6,000 x g for 15 minutes, with the supernatant carefully collected”.
- Tables 2 and 3, it is not clear if the values of protein in the band and protein per mL culture refer to total protein amount or to the specific recombinant proteins. How was protein per mL culture calculated?

**Our response:** Thank you for your insightful comment. To address your concern, we have clarified this issue in the manuscript under the subsection ‘Analysis of Total and Soluble Fusion Proteins’ in the Materials and Methods section. Specifically, the values of protein in the band and protein per mL culture refer to the specific recombinant proteins. The calculation of protein per mL culture was detailed in the revised subsection.

- L. 312, replace “conjugated” by “fused”

**Our response:** Thank you for pointing out this error. We have replace “conjugated” by “fused in the manuscript.

- L. 316-326, was the function analyzed by bioinformatics in this work? The comparison between the predicted and experimentally determined structures does not allow to infer the function of a protein. For this analysis, docking would be more appropriated. In this sense, “supporting” (L. 326) should be replaced by “suggesting”.

**Our response:** Thank you for your suggestion. We have replace “supporting” by “suggesting “in the manuscript.

- Table S1, please revise the table title that should be “Percentage of soluble recombinant proteins at different incubation temperatures, duration of incubation, and growth stages of IPTG induction.”

**Our response:** Thank you for your suggestion. We have corrected the title

- Tables S2 and S3, please clarify which are the unities of the values displayed on the tables, mg/L? μg/L? Are replicates performed for the statistical analysis?

**Our response:** Thank you for your comment. The units of protein in Tables S2 and S3 are represented as peak areas or grayscale intensity of the bands, which directly correlate with the amount of protein present. This information has been clarified in the manuscript.
