# Supplementary material for: Challenges and strategies in the soluble expression of CTA1-(S14P5)4-DD and CTA1-(S21P2)4-DD fusion proteins as candidates for COVID-19 intranasal vaccines
Source: PLoS One. 2024 Dec 26;19(12):e0306153. doi: 10.1371/journal.pone.0306153 (PMC11670946; doi:10.1371/journal.pone.0306153)
Supplement: Attachment — Submitted filename: Response to Reviewers.docx [file pone.0306153.s008.docx]

Dear Dr. Haitham Mohamed Amer,

We are grateful to the reviewers and the academic editor for their constructive feedback on our manuscript titled "Challenges and strategies in the soluble expression of CTA1-(S14P5)4-DD and CTA1-(S21P2)4-DD fusion proteins as candidates for COVID-19 intranasal vaccines" (PONE-D-24-23892R1). We have carefully considered all comments and have revised the manuscript accordingly. Below, we provide a point-by-point response to the reviewers’ comments.

**Journal Requirement: Reference List Review**

*Comment:* Please review your reference list to ensure that it is complete and correct. If you have cited papers that have been retracted, please include the rationale for doing so in the manuscript text, or remove these references and replace them with relevant current references.

*Response:* Thank you for reminding us. We have carefully reviewed and updated the reference list to ensure its accuracy and completeness. We have also checked for any retracted articles and confirm that none of the references used in our manuscript are retracted.

**Reviewer #3 Comment 2: Technical Soundness and Data Support**

*Comment:* The manuscript must describe a technically sound piece of scientific research with data that supports the conclusions. Reviewer #1: Yes; Reviewer #3: Partly.

*Response:* We appreciate the reviewer’s feedback and understand the importance of ensuring that the data presented fully support the conclusions drawn. Upon careful review, we confirm that the experiments were conducted with rigorous controls, appropriate replication, and sufficient sample sizes, all of which are detailed in the Methods section. We believe that the current data adequately support the conclusions, and no further revisions are necessary in this regard. However, we remain open to any specific suggestions from the reviewer to further improve clarity or robustness.

**Reviewer #3 Comment 6: Clarifications and Suggested References**

*Comment:* "The vaccines are expected to be thermostable because they are based on linear epitopes." The reviewer suggested that linear epitopes still may form different 3D structures and recommended including and discussing the following studies:

- Markosian, Christopher, et al. "Genetic and Structural Analysis of SARS-CoV-2 Spike Protein for Universal Epitope Selection."
- Staquicini, Daniela I., et al. "Design and Proof of Concept for Targeted Phage-Based COVID-19 Vaccination Strategies."

*Response:* We thank the reviewer for pointing out the need to clarify our statement regarding the thermostability of vaccines based on linear epitopes. We agree that linear epitopes can adopt different three-dimensional structures, and their stability can vary depending on their context.

**Revised Text:** We have rephrased the sentence in the Introduction as follows: "The inclusion of linear epitopes in the vaccine design may contribute to enhanced stability, as they are less reliant on specific tertiary structures for their antigenicity compared to conformational epitopes. However, the overall thermostability of the vaccine will depend on the properties of the entire fusion protein, including its folding and interactions between domains.”

Regarding the suggested references, we appreciate the reviewer’s effort to enhance our manuscript. However, after reviewing the articles by Markosian et al. and Staquicini et al., we believe that these studies are not directly relevant to our work:

- *Markosian et al. (2022)* focuses on epitope prediction and selection for broad vaccine coverage, whereas our work involves specific tandem repeat epitopes (S14P5 and S21P2) that have been previously validated for inducing neutralizing antibodies.
- *Staquicini et al. (2021)* discusses a phage-based vaccination strategy aimed at a cold-free supply chain, which focuses more on logistical aspects rather than the protein fusion strategy (CTA1-DD) for intranasal vaccines we are exploring.

Thus, while valuable, these references do not directly enhance the context of our study, and we have chosen not to include them.

**Reviewer #3 Comment: Hydrophobic Interaction Clarification**

*Comment:* "The authors also mentioned, 'Lowering temperature also diminishes the hydrophobic interaction between the expressed proteins.' Could the authors expand and clarify these effects?"

*Response:* We thank the reviewer for their suggestion to expand on the effects of lowering temperature on hydrophobic interactions during protein expression. We would like to note that this explanation is already included in the Discussion section of the manuscript (paragraph beginning with "The formation of insoluble expressed proteins as inclusion bodies emerged..."). This paragraph discusses how lowering the temperature reduces the kinetic energy of molecules, which diminishes the exposure of hydrophobic regions and reduces aggregation. We believe this explanation addresses the reviewer’s concern, but if further expansion is needed, we would be happy to provide additional clarification.

We hope that the revisions and responses provided meet the expectations of the reviewers and the editor. Thank you again for the opportunity to improve our manuscript. We look forward to your feedback.

Sincerely,

Simson Tarigan
Corresponding Author
Research Organization for Health, National Research and Innovation (BRIN), Indonesia
